# Defensive medicine practices and attitudes toward malpractice among physicians in Jordan: a cross-sectional study

**DOI:** 10.1186/s12913-026-15053-5

**Published:** 2026-06-30

**Authors:** Heba Ghozlan, Mohammed Baker, Nour Abdo, Anas J. Mistareehi, Lana Ma’adat, Dana Alnajjar, Husam Aldean Alqtami, Sewar Albaoull, Saba AL-Radaideh, Esra’a Abu-Zaitoun, Reema Al-Momani

**Affiliations:** 1 Department of Physiology and Biochemistry, Faculty of Medicine Jordan, University of Science and Technology, Irbid, Jordan; 2 Faculty of Medicine, Jordan University of Science and Technology, Irbid, Jordan; 3 Department of Public Health and Community Medicine, Faculty of Medicine, Jordan University of Science and Technology, Irbid, Jordan; 4 Department of Anatomy, Faculty of Medicine, Jordan University of Science and Technology, Irbid, Jordan

**Keywords:** Defensive medicine, Malpractice litigation, Physician attitudes, Healthcare policy, Medical liability, Legal literacy, Health services research, Healthcare utilization, Jordan

## Abstract

**Background:**

Defensive medicine (DM) refers to clinical practices motivated primarily by perceived medicolegal risk rather than patient benefit. In Jordan, evidence remains limited regarding physicians’ defensive practices, awareness of defensive medicine as a formal concept, understanding of the 2018 Medical Liability Law, and attitudes toward malpractice litigation.

**Methods:**

A cross-sectional survey was conducted among a convenience sample of 211 physicians in Jordan between August 2023 and March 2024. A structured questionnaire assessed DM practices (10-item scale), attitudes toward malpractice litigation (5-item scale), familiarity with defensive medicine, self-rated understanding of the 2018 Medical Liability Law, litigation exposure, insurance status, and perceived patient pressure. Multivariable linear regression analyses were performed to examine factors associated with defensive medicine and attitude scores.

**Results:**

Defensive medicine was reported by 84.4% of physicians, with a low-to-moderate overall intensity (mean item score across the 10 DM items : 2.1 ± 0.54 on the original 1–5 scale). Assurance behaviors predominated, particularly additional diagnostic testing perceived as precautionary or unnecessary (59.3%). Only 30.8% were familiar with the term “defensive medicine”, and 60.7% rated their understanding of the 2018 Medical Liability Law as poor. In the multivariable analysis, younger age (*B* = -0.34, *p* = 0.024), prior malpractice litigation experience (*B* = 4.89, *p* < 0.001), and perceiving litigation as a personal attack (*B* = -1.18, *p* = 0.012) were independently associated with higher defensive medicine scores (*R*² = 0.178). Specialty risk category was not independently associated with defensive medicine after adjustment. In the attitude model, familiarity with defensive medicine (*B* = 0.82, *p* = 0.028) and lower defensive medicine scores (*B* = -0.06, *p* = 0.047) were associated with higher attitude scores toward malpractice litigation (*R*² = 0.132).

**Conclusion:**

Defensive medicine was common among physicians in this sample and was expressed mainly through assurance-type behaviors. The findings suggest that defensive practices may be more closely associated with perceived professional vulnerability, prior litigation experience, and threat-related perceptions than with specialty risk category or self-rated legal understanding alone. Given the cross-sectional design and convenience sampling, these findings should be interpreted as exploratory and require confirmation in larger, more representative studies.

**Supplementary Information:**

The online version contains supplementary material available at https://doi.org/10.1186/s12913-026-15053-5.

## Introduction

Defensive medicine (DM) was first formally described in the United States in the early 1970s and refers to clinical practices motivated primarily by the desire to reduce physicians’ legal liability rather than to optimize patient care [1–3]. These practices are conventionally categorized into two distinct behavioral patterns: assurance behaviors, which involve providing additional services such as ordering extra tests, requesting superfluous consultations, or performing unnecessary procedures, and avoidance behaviors, which are characterized by refusing high-risk procedures or declining to treat high-risk patients [4, 5]. Although intended to reduce malpractice risk, DM contributes to escalating healthcare costs, exposes patients to unnecessary risks, and creates substantial inefficiencies in healthcare delivery [6–8].

Fear of malpractice litigation has been consistently identified as a major driver of defensive practices, with particularly high prevalence reported in high-risk medical specialties such as obstetrics, surgery, and emergency medicine [9–11].

The prevalence and correlates of DM vary considerably across countries, reflecting differences in medicolegal systems, cultural norms, and healthcare structures [12, 13]. International evidence shows considerable heterogeneity, ranging from moderate levels in countries with established tort reform to markedly higher rates in settings with emerging or recently reformed liability frameworks [5, 14–16]. Recent systematic reviews indicate that defensive practices may arise not only from direct litigation concerns but also from broader professional and systemic pressures, including patient expectations and concerns about reputation and accountability [13, 17]. From a health services research perspective, DM represents an important source of low-value care because it may increase unnecessary resource utilization, inflate system costs, and compromise care quality without commensurate patient benefit.

In Jordan, the enactment of the 2018 Medical Liability Law represented an important step toward establishing a formal framework for malpractice accountability while protecting both patients and physicians [18, 19]. The law aimed to unify and clarify the legal framework surrounding medical liability, which had previously been fragmented across multiple legal instruments [18]. However, Jordan continues to operate within a mixed medicolegal environment that combines civil liability principles with administrative oversight through specialized medical committees, creating a dual framework that may generate professional uncertainty for physicians navigating these parallel accountability structures [19, 20]. Within this environment, physicians continue to express concern about rising litigation risk and limited legal protection [20]. A prior national study documented a high prevalence of DM, with fear of malpractice and patient pressure identified as important contributing factors [21]. However, that work focused mainly on prevalence and broad correlates. Important gaps remain regarding physicians’ awareness of DM as a formal concept, their self-rated understanding of the 2018 Medical Liability Law, and the attitudinal dimensions underlying these behaviors. In particular, little is known about how physicians perceive malpractice litigation emotionally and professionally, and whether these perceptions are associated with defensive practices across demographic and specialty subgroups. By addressing these questions, the present study extends prior Jordanian work beyond prevalence estimates alone and provides new evidence on the medicolegal and attitudinal context in which DM occurs in Jordan.

This study was informed by established behavioral frameworks emphasizing the role of attitudes, alongside social and contextual influences, in shaping clinical decision-making [22]. In the context of DM, physicians’ attitudes toward malpractice litigation, including both threat perceptions and recognition of potential benefits, may influence defensive responses [23–25]. At the same time, demographic and professional characteristics such as medical specialty, years of clinical experience, sex, and prior personal or vicarious litigation exposure may also be associated with both attitudes and defensive practices. Evidence from other Middle Eastern settings has suggested that litigation experience, specialty risk profiles, and generational differences may be associated with DM engagement [26, 27]. However, these relationships remain underexplored in Jordan.

This study aims to provide a comprehensive analysis of DM practices among physicians in Jordan. Specifically, it aims to (1) assess physicians’ awareness of DM and the 2018 Medical Liability Law, (2) characterize the prevalence, types, and patterns of defensive practices across specialty and demographic subgroups, and (3) examine physicians’ attitudes toward malpractice litigation and their association with defensive behavioral practices.

## Materials and methods

### Study design

This cross-sectional study was conducted from August 2023 to March 2024 to estimate the prevalence of DM behaviors, assess physicians’ attitudes toward malpractice litigation, and identify associated factors within the framework of Jordan’s 2018 Medical Liability Law. Data were collected via a self-administered questionnaire completed either online or on paper in selected hospital settings.

### Study population and sampling

The targeted population included physicians actively practicing in Jordan and licensed by the Jordanian Ministry of Health or equivalent authorities. Eligible participants included interns, general practitioners, physicians in postgraduate specialty training programs (years 1–6), board-certified specialists, and consultants working across multiple sectors, including the Ministry of Health, the Royal Medical Services, university hospitals, and private institutions. Physicians practicing outside Jordan and non-physician healthcare workers were excluded. Because recruitment relied on open dissemination through professional social media networks and selected hospital-based distribution, no closed or enumerated sampling frame was available.

A non-probability convenience sampling approach was used. The online questionnaire link was disseminated through professional networks, including physician-specific Facebook and WhatsApp groups, allowing voluntary participation from physicians across different regions and sectors. In addition, paper-based questionnaires were distributed directly by the research team to physicians in selected hospitals who were available and willing to participate. No random or stratified sampling was performed. Because the online survey link could be freely shared, the number of physicians who received or viewed the invitation could not be determined; therefore, a conventional response rate could not be calculated. Duplicate participation could not be completely excluded, although responses were screened before analysis to identify obvious duplicates and incomplete submissions.

The required sample size was estimated using a single-proportion formula with finite-population correction, based on an approximate national physician population of 44,000, an expected DM prevalence of 80%, a 95% confidence level, and a 5% margin of error, yielding a target sample size of approximately 245 physicians. A total of 211 physicians were included in the final analysis.

### Data collection instrument

Data were collected using a structured questionnaire consisting of six sections addressing (1) sociodemographic and professional characteristics (2), DM practices, (3) awareness and perceived legal knowledge, (4) triggering and contextual factors, (5) attitudes toward malpractice litigation, and (6) patient-perceived pressure for unnecessary care. The complete questionnaire is provided in Additional File 1.

The questionnaire was translated from English to Arabic using a forward-backward translation process, with discrepancies resolved by consensus to ensure semantic equivalence. Before full deployment, the instrument was pilot tested among practicing physicians to assess clarity, relevance, and contextual appropriateness, and minor wording adjustments were made accordingly. However, no formal psychometric validation was conducted in the Jordanian population, and the instrument should therefore be interpreted as a context-adapted survey rather than a newly validated tool.

The first section obtained information on participants’ age (continuous variable; also categorized as 23–30, 31–40, > 40 years), sex, years of experience (continuous variable; also categorized as 1–5, 6–10, 11–20, > 20 years), current medical position (intern/general practitioner, resident, specialist, or consultant), medical specialty, employment sector (Ministry of Health, Royal Medical Services, university hospitals, or private sector), geographic region of practice (North, Central, or South Jordan), and average weekly working hours (categorized as < 72, 72–130, > 130 h).

Medical specialties were grouped into high-, moderate-, and low-risk categories using a modified a priori classification based on published literature and contextual adaptation for Jordan (Additional File 2, Table S2). This approach was informed by evidence that malpractice risk varies substantially across specialties, with procedural and surgical fields generally showing the highest overall claim exposure [11]. Dermatology was classified as moderate risk because, although it is generally associated with lower overall claim frequency than classic high-risk specialties, published medicolegal literature identifies recurring liability related to procedural dermatology, delayed diagnosis of skin cancer, failure to biopsy suspicious lesions, and cosmetic or operative complications [28, 29]. Pediatrics was also classified as moderate risk because, although overall claim frequency is lower than in the highest-risk specialties, pediatric practice remains medicolegally sensitive because of diagnostic uncertainty and the potentially high severity of claims involving permanent injury [30, 31]. Because no universally accepted Jordan-specific malpractice-risk classification exists, this grouping should be interpreted as a pragmatic contextual categorization rather than a definitive external standard.

The second section measured DM practices using a 10-item DM Scale originally developed and validated by Ünal & Akbolat in a Turkish sample of physicians [32]. The scale included two domains: assurance behaviors (6 items) and avoidance behaviors (4 items). Each item was rated on a 5-point Likert scale ranging from 1 (“never”) to 5 (“always”). In the present study, the scale demonstrated good internal consistency (Cronbach’s α = 0.82). Participants reported behaviors they personally perceived as unnecessary or precautionary. As no standardized clinical criteria or objective verification methods (e.g., chart review or utilization data) were applied, responses reflect subjective perceptions of “unnecessary” care. Total DM scores ranged from 10 to 50. For descriptive presentation, scores were also expressed as mean item values on the original 1–5 scale, with higher values indicating greater engagement in defensive practices. No established cut-off values exist for this scale to categorize low, moderate, or high DM engagement. Item-level responses were also analyzed descriptively.

The third section assessed three dimensions of awareness and perceived legal knowledge: (1) prior familiarity with the concept of DM (yes/no), (2) perceived increase in malpractice litigation cases in recent years (yes/no), and (3) self-rated understanding of the 2018 Medical Liability Law, assessed on a 5-point Likert scale ranging from 1 (“very poor understanding”) to 5 (“excellent understanding”). This measure reflects physicians’ perceived understanding rather than objectively tested legal knowledge.

The fourth section examined contextual factors associated with DM behaviors. Participants were asked whether they had ever been personally involved in a malpractice lawsuit (yes/no), whether any of their colleagues had been subject to litigation to their knowledge (yes/no), and their perception of liability insurance coverage adequacy, categorized as absent, inadequate, or adequate.

The fifth section measured physicians’ attitudes toward malpractice litigation using a 5-item scale adapted from a previously validated study by Renkema et al. [23]. The original scale was developed among physicians in the Netherlands. The adapted scale captures both negative and positive attitudinal dimensions. Items 1–3 reflect negative attitudes: (1) malpractice lawsuits are harmful to a physician’s reputation and self-confidence; (2) malpractice lawsuits cause stress and evoke guilt; and (3) malpractice lawsuits are perceived as a personal attack. Items 4–5 reflect positive attitudes: (4) malpractice lawsuits are a form of justice, and (5) malpractice lawsuits are a way to improve the quality of care. Each item was scored on a 5-point Likert scale from 1 (“strongly disagree”) to 5 (“strongly agree”). To generate a composite attitude score, items 1–3 were reverse-coded so that higher total scores indicated less negative and more accepting attitudes toward malpractice litigation. Total attitude scores ranged from 5 to 25. Both total scores and individual item responses were analyzed.

The final section assessed patients’ perceived pressure to provide unnecessary care. Participants were asked to complete the statement: “When patients request further examination or treatment, I feel pressured to ———— even if it is clinically not strictly necessary.” They could select one or more responses, including performing further examinations or investigations, prescribing medications or treatments, referring the patient to another center, or not feeling pressured to act.

### Survey administration

The questionnaire was administered in two formats: an online version created using Google Forms and a paper-based version used in selected hospital settings. The first page of both formats presented the study’s objectives, ethical approval information, a voluntary participation statement, and confidentiality assurances. Only participants who provided consent could proceed to the questionnaire. No identifying personal information was collected. All responses were stored securely and later exported for statistical analysis.

### Ethical considerations

Ethical approval for this study was obtained from the Institutional Review Board (IRB) of Jordan University of Science and Technology (JUST) and King Abdullah University Hospital (KAUH) (Ref. 65/162/2023) before data collection. The study was conducted in accordance with the ethical principles of the Declaration of Helsinki. Participation was entirely voluntary, informed consent was obtained from all participants, and no identifying information was collected. Data confidentiality and anonymity were maintained throughout the study.

### Statistical analysis

Data were analyzed using SAS software version 9.4 (SAS Institute Inc., Cary, NC, USA). Descriptive statistics were computed to summarize participants’ characteristics and study variables. Categorical variables were presented as frequencies and percentages. Continuous and ordinal variables were reported as means with standard deviations (SD) or medians with interquartile ranges (IQR), as appropriate.

For Likert-type outcomes, item-level responses were treated as ordinal for descriptive purposes. However, the composite multi-item scores for the total DM score and total attitude score were summarized using means and SDs to aid interpretability because these scores represent aggregated responses across multiple items. Continuous variables such as age and years of experience were analyzed as continuous measures in inferential analyses, including Spearman correlations and multivariable linear regression, to preserve statistical power and assess linear trends. However, they were also categorized in descriptive tables to facilitate clinical interpretation and comparison with prior studies.

Normality of the composite outcome scores was assessed using the Shapiro-Wilk test. Because both the total DM score and the total attitude score were non-normally distributed, non-parametric methods were used for bivariate analyses. The Mann-Whitney U test was used for comparisons between two groups, and the Kruskal-Wallis H test was used for comparisons across three or more groups. When the Kruskal-Wallis test was statistically significant, post hoc pairwise comparisons were performed using the Dwass-Steel-Critchlow-Fligner procedure. Associations between continuous or ordinal variables were examined using Spearman’s rank correlation coefficient (*ρ*). Effect sizes were reported for bivariate comparisons using Spearman’s ρ for correlations, Cohen’s d for two-group comparisons, and eta-squared (η²) for Kruskal-Wallis tests; a full summary is provided in Additional File 2, Table S6.

To examine adjusted associations, multivariable linear regression models were fitted with the total DM score and the total attitude score as dependent variables, respectively. Although these scores were derived from ordinal Likert-type items, they were analyzed as a continuous composite measure because each score summarized responses across multiple items. Candidate predictors were selected based on bivariate associations (*p* < 0.20) and a priori clinical and contextual relevance, and included demographic, professional, awareness, and medicolegal variables. Multicollinearity was assessed using variance inflation factors (VIFs), and model fit was evaluated using the F-statistic, *R*², and adjusted *R*². Because age and years of experience are conceptually related, both variables were initially considered as continuous indicators of career stage; collinearity diagnostics showed acceptable VIF values (DM model: 1.005–1.024; attitude model: 1.051–1.130), indicating no evidence of problematic multicollinearity. Regression tables report unstandardized coefficients (*B*), standard errors (SE), and 95% confidence intervals (CI). A parallel exploratory stepwise regression (entry/stay *p* < 0.15) was also performed for both outcomes and is presented in Additional File 2, Tables S7–S8. These stepwise models were treated as secondary, hypothesis-generating analyses. All statistical tests were two-tailed. Exact p-values are reported to three decimal places where possible; values < 0.001 are reported as *p* < 0.001. A p-value < 0.05 was considered statistically significant.

## Results

### Participant demographics and clinical characteristics

Table 1 summarizes the demographic and professional characteristics of the 211 participating physicians. The sample was predominantly young and early-career, with a mean age of 30.0 ± 6.2 years; 73.9% were aged 23–30 years. Most participants were residents (60.7%) and hospital-based physicians, particularly from university hospitals (44.6%) and Royal Medical Services hospitals (23.7%). Surgical and medical specialties represented the largest specialty groups. More than half of participants (55.4%) reported working 72–130 hours per week. Overall, the sample was concentrated in younger, hospital-based, and early-career physicians.

**Table 1:** Demographic and professional characteristics of the participating physicians (*N* = 211)

Characteristic	Category / Statistic	*n* (%) / Value
Sex	Male	118 (55.9)
	Female	93 (44.1)
Age	Mean ± SD	30.0 ± 6.2
	Median (IQR)	28.0 (27.0–31.0)
	23–30	156 (73.9)
	31–40	42 (19.9)
	> 40	13 (6.2)
Current professional position	General Practitioner	39 (18.5)
	Resident (Years 1–6)	128 (60.7)
	Specialist	33 (15.6)
	Consultant	11 (5.2)
Professional experience	Mean ± SD	5.5 ± 6.0
	Median (IQR)	4.0 (2.0–6.0)
	1–5 years	145 (68.7)
	6–10 years	47 (22.3)
	11–20 years	13 (6.2)
	> 20 years	6 (2.8)
Specialty category	Surgical specialties	75 (35.5)
	Medical specialties	74 (35.1)
	Primary care	45 (21.3)
	Diagnostic & others	17 (8.1)
Employment sector	University hospitals	94 (44.6)
	Royal Medical Services	50 (23.7)
	Ministry of Health	41 (19.4)
	Private sector	26 (12.3)
Geographic region	North Jordan	145 (68.7)
	Central Jordan	60 (28.4)
	South Jordan	6 (2.8)
Weekly working hours	< 72	73 (34.6)
	72–130	117 (55.4)
	> 130	21 (10.0)

Data are presented as *n* (%), mean ± SD, or median (IQR), as appropriate. Percentages may not total 100% due to rounding. Age is reported in years, and professional experience is reported in years since graduation/practice, as applicable. Specialty groupings are provided for analytical purposes; full specialty distribution is presented in Additional File 2, Table S1

### Physician awareness and knowledge of the medicolegal context

Physicians demonstrated limited awareness of the formal concept of DM, with only 30.8% reporting prior familiarity with the term (Table 2). In contrast, most participants (87.2%) perceived that malpractice litigation had increased in recent years. Self-reported understanding of the Jordanian Medical Liability Law of 2018 was generally low: 60.7% rated their understanding as poor, whereas only 15.6% rated it as good-to-excellent. The mean self-rated understanding score was 2.26 ± 1.12 on a 5-point scale, indicating limited perceived medicolegal understanding in this sample.

**Table 2:** Physician awareness and perceptions of the medicolegal environment (*N* = 211)

Variable	Category	*n* (%)
Heard of Defensive Medicine	Yes	65 (30.8)
	No	146 (69.2)
Perceived Increase in Malpractice Litigation	Yes	184 (87.2)
	No	27 (12.8)
Self-Rated Understanding of the 2018 Medical Liability Law	Poor (1–2)	128 (60.7)
	Moderate (3)	50 (23.7)
	Good-Excellent (4–5)	33 (15.6)

Data are presented as *n* (%), unless otherwise indicated. Self-rated understanding of the 2018 Medical Liability Law was assessed on a 5-point Likert scale (1 = very poor, 5 = excellent) and categorized as poor (1–2), moderate (3), and good-to-excellent (4–5). Percentages are based on the total sample (N *=* 211)

### Triggering factors for defensive medicine

Several contextual factors potentially associated with DM were identified (Table 3).

**Table 3:** Potential triggers of defensive medical behavior (*N* = 211)

Factor	Category	*n* (%)
Personal Lawsuit Experience	No	195 (92.4)
	Yes	16 (7.6)
Colleague Lawsuit Experience	No	129 (61.1)
	Yes	82 (38.9)
Liability Insurance Status	No insurance	108 (51.2)
	Inadequate coverage	60 (28.4)
	Adequate coverage	43 (20.4)
Patient Pressure Responses	Additional examinations/investigations	76 (37.4)
	Prescribe medications/treatments	41 (20.2)
	Refer to another facility	18 (8.9)
	No pressure reported	108 (53.2)

Data are presented as *n* (%). Patient pressure responses were non-mutually exclusive, and participants could select more than one option. Percentages for patient-pressure response items were calculated based on *n* = 203 after excluding inconsistent responses from participants who selected both “no pressure” and one or more pressure-related actions. Therefore, percentages for these items may sum to more than 100%

Direct personal experience with malpractice litigation was relatively uncommon, reported by 7.6% of participants, although indirect exposure through colleagues was more frequent (38.9%). Descriptive variation across specialty-risk groups was observed, with higher proportions of personal litigation experience in high-risk and moderate-risk groups than in the low-risk group (Table 4), although these subgroup comparisons should be interpreted cautiously because of small numbers.

**Table 4:** Personal malpractice litigation experience by specialty-risk group

Specialty Group	Specialty	Total (*n*)	Physicians with lawsuits, *n* (%)
High risk	General Surgery	35	3 (8.6)
	Orthopedics/Trauma	9	2 (22.2)
	Neurosurgery	8	2 (25.0)
	Obstetrics & Gynecology	6	1 (16.7)
	Anesthesia	17	1 (5.9)
	**Subtotal**	**75**	**9 (12.0)**
Moderate risk	Internal Medicine	18	1 (5.6)
	Pediatrics	13	2 (15.4)
	Dermatology	12	2 (16.7)
	**Subtotal**	**43**	**5 (11.6)**
Low risk	General Practice	35	1 (2.9)
	Family Medicine	10	1 (10.0)
	**Subtotal**	**45**	**2 (4.4)**
Total	—	**211**	**16 (7.6)**

Data are presented as *n* (%). Percentages are calculated within each specialty or specialty-risk group. The specialty-risk classification was defined a priori, informed by published literature, and adapted for the study context; details are provided in Additional File 2, Table S2. The total incidence (16/211, 7.6%) was calculated across the full study sample, including specialties not individually shown in this table

Perceived financial vulnerability was also common. More than half of the participants lacked liability insurance, and only 20.4% reported feeling adequately protected. Patient-related pressures were also reported, particularly pressure to provide additional examinations, investigations, medications, or referrals that physicians may not otherwise have considered necessary.

Taken together, these findings indicate that medicolegal concerns, perceived insurance inadequacy, and patient expectations coexist within the practice environment and may represent relevant contextual correlates of defensive medical behaviors.

### Prevalence and patterns of defensive medicine behaviors

Self-reported DM behaviors were commonly observed in this sample of Jordanian physicians. The overall mean DM score was 2.1 ± 0.54 (mean item score across the 10 DM items on the original 1–5 scale, where 1 = never and 5 = always). Overall, 84.4% (*n* = 178) of participants reported engaging in at least one defensive behavior that they perceived as precautionary or unnecessary sometimes or more frequently (Table 5).

**Table 5:** Frequency distribution of self-reported defensive medicine behaviors (*N* = 211)

DM Behavior	Never *n* (%)	Rarely *n* (%)	Sometimes *n* (%)	Usually *n* (%)	Always *n* (%)	Mean ± SD
**Assurance Behaviors**						
1. Ordering unnecessary diagnostic tests	22 (10.4%)	64 (30.3%)	84 (39.8%)	36 (17.1%)	5 (2.4%)	2.71 ± 0.95
2. Ordering unnecessary screenings	24 (11.4%)	82 (38.9%)	83 (39.3%)	21 (10.0%)	1 (0.5%)	2.49 ± 0.84
3. Requesting unnecessary consultations	57 (27.0%)	94 (44.6%)	44 (20.8%)	16 (7.6%)	0 (0.0%)	2.09 ± 0.88
4. Hospitalizing patients without necessity	96 (45.5%)	79 (37.4%)	31 (14.7%)	5 (2.4%)	0 (0.0%)	1.74 ± 0.79
5. Prescribing unnecessary medications	77 (36.5%)	92 (43.6%)	33 (15.6%)	8 (3.8%)	1 (0.5%)	1.88 ± 0.84
6. Performing unnecessary invasive procedures	136 (64.5%)	56 (26.5%)	14 (6.6%)	4 (1.9%)	1 (0.5%)	1.47 ± 0.74
**Avoidance Behaviors**						
7. Referring patients to other departments/ hospitals	46 (21.8%)	72 (34.1%)	77 (36.5%)	16 (7.6%)	0 (0.0%)	2.30 ± 0.89
8. Taking initiative less frequently	68 (32.2%)	84 (39.8%)	50 (23.7%)	8 (3.8%)	1 (0.5%)	2.00 ± 0.87
9. Avoiding treatment of high-risk patients	64 (30.3%)	72 (34.1%)	53 (25.1%)	20 (9.5%)	2 (1.0%)	2.16 ± 1.00
10. Avoiding new treatment methods	65 (30.8%)	75 (35.6%)	58 (27.5%)	10 (4.7%)	3 (1.4%)	2.10 ± 0.94

Responses were recorded on a 5-point Likert scale (1 = Never, 5 = Always). Data are presented as *n* (%) for each response category and mean ± SD for each item. Percentages are based on *N* = 211 for each item. The overall mean score represents the mean item score across all 10 DM behaviors, with higher values indicating more frequent self-reported engagement in DM practices. Items reflect physicians’ self-reported perceptions of precautionary or defensive practices and should not be interpreted as objectively verified unnecessary care

Assurance behaviors predominated, particularly the ordering of additional diagnostic tests that respondents perceived as precautionary or unnecessary (59.3%). Among assurance behaviors, ordering additional diagnostic testing had the highest mean score (2.71 ± 0.95), followed by ordering additional screening tests (2.49 ± 0.84). By contrast, invasive procedures perceived as unnecessary or precautionary were the least frequently reported assurance behavior (1.47 ± 0.74).

Avoidance behaviors were also reported, but generally at lower levels. Within this domain, referring patients to other departments or hospitals was the most frequently reported avoidance behavior (2.30 ± 0.89), whereas practices such as taking initiative less frequently, avoiding treatment of high-risk patients, and avoiding new treatment methods, were less commonly reported.

Overall, DM in this sample was characterized predominantly by assurance-type behaviors.

### Physicians’ attitudes toward malpractice litigation

Physicians’ attitudes toward malpractice litigation reflected a mixed pattern, combining perceived emotional burden with recognition of potential professional value (Table 6). Approximately half agreed that malpractice litigation negatively affects physicians’ professional reputation and self-confidence and causes stress and feelings of guilt. By contrast, fewer participants perceived litigation as a personal attack, although many expressed neutral views on this item.

**Table 6:** Physicians’ attitudes toward malpractice litigation (*N* = 211)

Item	Strongly Disagree *n* (%)	Disagree *n* (%)	Neutral *n* (%)	Agree *n* (%)	Strongly Agree *n* (%)	Mean ± SD	
1. Harmful for the reputation and self-confidence	7 (3.3)	43 (20.4)	56 (26.5)	84 (39.8)	21 (10.0)		2.67 ± 1.02
2. Causes stress and feelings of guilt	7 (3.3)	36 (17.1)	62 (29.4)	90 (42.6)	16 (7.6)		2.66 ± 0.96
3. Perceived as a personal attack	22 (10.4)	75 (35.5)	77 (36.5)	28 (13.3)	9 (4.3)		3.34 ± 0.98
4. Represents a form of justice	6 (2.8)	40 (19.0)	71 (33.7)	79 (37.4)	15 (7.1)		3.27 ± 0.94
5. Improves quality of care	12 (5.7)	25 (11.9)	73 (34.6)	79 (37.4)	22 (10.4)		3.35 ± 1.01

Responses were recorded on a 5-point Likert scale (1 = strongly disagree, 5 = strongly agree). Data are presented as *n* (%) and mean ± SD. For interpretive consistency, items 1–3 were reverse-scored for calculation of the composite attitude score, such that higher values indicate less negative and more accepting attitudes toward malpractice litigation. Percentages are based on *N* = 211

At the same time, substantial proportions acknowledged potentially constructive aspects of malpractice litigation, including its role as a mechanism of justice and as a possible contributor to quality improvement. The overall mean attitude score was 3.06 ± 0.46 on the 5-point scale, indicating generally balanced attitudes toward malpractice litigation, with higher scores reflecting less negative attitudes after reverse-coding of items 1–3.

### Factors associated with defensive medicine practices and attitudes toward litigation

#### Factors associated with defensive medicine

In bivariate analyses, younger age was significantly associated with higher DM scores (Spearman’s *ρ* = -0.230, *p* < 0.001). DM scores also differed across specialty-risk categories (Kruskal-Wallis *H* = 6.933, *p* = 0.031). Post-hoc Dwass-Steel-Critchlow-Fligner comparisons showed that physicians in moderate-risk specialties had lower scores than those in low-risk specialties (*p* = 0.045), whereas the other pairwise differences were not statistically significant (Additional File 2, Table S4). Physicians with personal experience of malpractice litigation also had higher DM scores (*p* = 0.008). No statistically significant bivariate associations were observed for sex, professional experience, healthcare sector, or geographic region (Table 7). Overall, the bivariate effect sizes for DM were small (Additional File 2, Table S6).

**Table 7:** Associations between physician characteristics and defensive medicine and attitude scores

Factor Group	Specific Factor	Test Statistic (DM)	*p*-value (DM)	Test Statistic (Attitude)	*p*-value (Attitude)
Demographics	Sex	*U* = 9828.5	0.946	U = 10902.5	0.016*
	Age	*ρ* = -0.23	< 0.001*	*ρ* = -0.150	0.029*
Professional	Specialty risk	*H* = 6.933	0.031*	*H* = 1.280	0.527
	Professional experience	*ρ* = -0.113	0.100	*ρ* = -0.131	0.057
	Sector	*H* = 3.283	0.350	*H* = 4.954	0.175
	Geographic region	*H* = 0.138	0.933	*H* = 5.682	0.058
Awareness	Heard of defensive medicine	*U* = 6651.5	0.560	*U* = 7731.5	0.037*
	Perceived Increase in Malpractice Litigation	*U* = 3058.5	0.506	*U* = 3026	0.574
	Self-Rated Understanding of the 2018 Medical Liability Law	*ρ* = -0.0278	0.688	*ρ* = -0.070	0.313
Triggering factors	Personal lawsuit experience	*U* = 2313.5	0.008*	*U* = 1402.5	0.205
	Colleague’s lawsuit experience	*U* = 8764	0.867	*U* = 9057.5	0.391
	Insurance adequacy	*H* = 2.434	0.296	*H* = 3.900	0.142

*U* = Mann-Whitney *U* statistic; *H* = Kruskal-Wallis *H* statistic; *ρ* = Spearman’s rank correlation coefficient. Statistically significant associations (*p* < 0.05) are indicated with asterisks (*). Median (IQR) subgroup distributions are presented in Additional File 2, Tables S3 and S5

In the multivariable linear regression analysis (Table 8), younger age (*B* = − 0.34, *p* = 0.024), personal experience of malpractice litigation (*B* = 4.89, *p* < 0.001), and stronger perception of litigation as a personal attack (*B* = − 1.18, *p* = 0.012) were independently associated with higher DM scores. Other variables, including sex, professional experience, specialty risk category, familiarity with DM, perceived increase in litigation, self-rated understanding of the Medical Liability Law, colleague litigation experience, insurance adequacy, and overall attitude score, were not independently associated with DM score. The model explained 17.8% of the variance in DM scores (*R*² = 0.178, adjusted *R*² = 0.128).

**Table 8:** Multivariable linear regression analysis of factors associated with defensive medicine score

Variable	B	SE	95% CI	*p*-value
Intercept	35.28	5.07	25.28, 45.29	< 0.001
Sex	-0.46	0.74	-1.93, 1.01	0.540
Age	-0.34	0.15	-0.63, -0.05	0.024*
Specialty risk group	-0.51	0.47	-1.43, 0.41	0.274
Professional experience	0.14	0.15	-0.16, 0.44	0.345
Familiarity with DM	0.71	0.86	-0.99, 2.42	0.410
Perceived increase in malpractice litigation	0.14	1.10	-2.04, 2.31	0.900
Self-rated understanding of 2018 Medical Liability Law	0.16	0.36	-0.56, 0.88	0.658
Personal litigation experience	4.89	1.40	2.13, 7.66	< 0.001*
Colleague litigation experience	0.04	0.74	-1.43, 1.51	0.954
Insurance adequacy	-0.56	0.52	-1.58, 0.47	0.285
Overall attitude score	-0.03	0.20	-0.43, 0.36	0.871
Perception of litigation as personal attack	-1.18	0.46	-2.09, -0.26	0.012*

Model summary: R² = 0.178, adjusted R² = 0.128, F(12, 198) = 3.57, *p* < 0.001. Coefficients are unstandardized (B). The item “litigation is perceived as a personal attack” was reverse-coded before analysis; therefore, higher scores indicate a lower perceived personal threat. Statistically significant coefficients (*p* < 0.05) are marked with an asterisk (*)

In the exploratory stepwise regression (Additional File 2, Table S7), younger age, personal malpractice litigation experience, and perceiving litigation as a personal attack were retained in the final model. This model explained 16.3% of the variance in DM score (*R²* = 0.163, adjusted *R²* = 0.151; F = 13.45, *p* < 0.001). All retained predictors remained statistically significant.

#### Factors associated with attitudes toward malpractice litigation

In bivariate analyses, female physicians had higher attitude scores than male physicians (*p* = 0.016). Younger age was also associated with higher attitude scores (Spearman’s *ρ* = -0.150, *p* = 0.029), and physicians familiar with the concept of DM had higher attitude scores (*p* = 0.037). No significant bivariate associations were observed for specialty risk category, professional experience, healthcare sector, geographic region, personal or colleague litigation exposure, perceived insurance adequacy, or perceived increase in malpractice litigation (Table 7). Most bivariate effect sizes for attitude were small (Additional File 2, Table S6).

The correlation between total DM and total attitude scores was not statistically significant (Spearman’s *ρ* = -0.117, *p* = 0.091) (Table 9). At the item level, however, perceiving litigation as a personal attack was associated with higher DM scores (Spearman’s *ρ* = -0.222, *p* = 0.001). The remaining attitude items were not significantly correlated with DM scores.

**Table 9:** Correlation between defensive medicine scores and attitude scores

Category	Variable / Item Description	Spearman ρ	*p*-value
Overall Correlation	DM Score vs. Overall Attitude Score	-0.117	0.091
Item-level Correlations	Harmful for the reputation and self-confidence	-0.129	0.062
	Causes stress and feelings of guilt	-0.028	0.684
	Perceived as a personal attack	-0.222	0.001*
	Represents a form of justice	0.124	0.073
	Improves quality of care	0.018	0.797

Spearman’s rank correlation is used for all associations. Statistically significant associations (*p* < 0.05) are indicated with asterisks (*)

In the multivariable linear regression analysis (Table 10), familiarity with DM was independently associated with higher attitude scores toward malpractice litigation (*B* = 0.82, *p* = 0.028), whereas higher DM scores were independently associated with lower attitude scores (*B* = − 0.06, *p* = 0.047). After adjustment, age and sex were no longer statistically significant. The model explained 13.2% of the variance in attitude scores (*R*² = 0.132, adjusted *R*² = 0.084).

**Table 10:** Multivariable linear regression analysis of factors associated with attitude score

Variable	B	SE	95% CI	*p*-value
Intercept	19.01	2.03	15.00, 23.02	< 0.001
Sex	0.45	0.32	-0.18, 1.08	0.160
Age	-0.07	0.06	-0.20, 0.05	0.246
Specialty risk group	-0.12	0.20	-0.52, 0.27	0.538
Professional experience	-0.01	0.07	-0.13, 0.13	0.987
Familiarity with DM	0.82	0.37	0.09, 1.54	0.028*
Perceived increase in malpractice litigation	-0.19	0.48	-1.13, 0.75	0.693
Self-rated understanding of 2018 Medical Liability Law	-0.24	0.16	-0.55, 0.07	0.127
Personal litigation experience	-0.94	0.62	-2.16, 0.29	0.133
Colleague litigation experience	0.42	0.32	-0.21, 1.05	0.187
Insurance adequacy	0.28	0.22	-0.16, 0.72	0.209
DM score	-0.06	0.03	-0.12, -0.001	0.047*

Model summary for attitude score: *R*² = 0.132, adjusted *R*² = 0.084, F(11, 199) = 2.74, *p* = 0.003. Coefficients are unstandardized (*B*). Higher attitude scores indicate less negative and more accepting attitudes toward malpractice litigation after reverse- coding of negatively phrased items (items 1–3). Statistically significant coefficients (*p* < 0.05) are marked with an asterisk (*)

In the exploratory stepwise regression (Additional File 2, Table S8), age, DM score, familiarity with the concept of DM, personal malpractice litigation experience, and sex were retained under the entry/stay criterion. The final model explained 10.9% of the variance in attitude score (*R²* = 0.109, adjusted *R²* = 0.088; F = 5.04, *p* < 0.001). In this model, younger age and familiarity with DM remained associated with higher attitude scores, whereas higher DM scores remained associated with lower attitude scores. Personal malpractice litigation experience and sex were retained in the model but were not statistically significant.

Overall, the exploratory stepwise analyses were broadly consistent with the primary adjusted models and also indicated modest explanatory power. These findings should be interpreted as supplementary and hypothesis-generating rather than definitive.

## Discussion

**Fig. 1:**
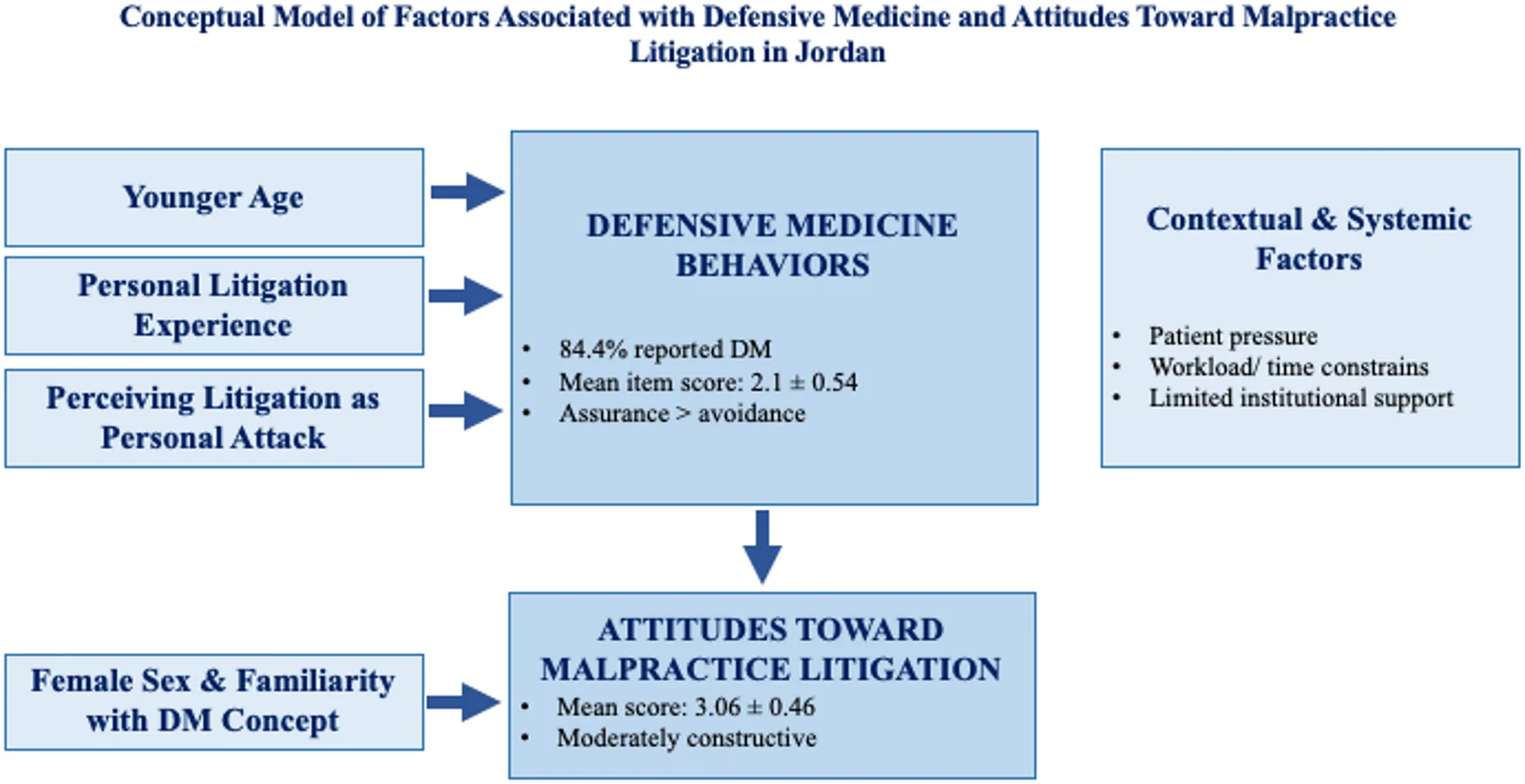
Conceptual summary of the independently associated variables identified in the multivariable regression analyses and relevant contextual considerations. Solid arrows represent statistically independent associations from the adjusted models. The contextual and systemic factors box summarizes interpretive considerations discussed in the text, and does not represent a modeled or causal pathway

This study provides one of the first multidimensional assessments of DM in Jordan after the implementation of the 2018 Medical Liability Law. It extends prior Jordanian research beyond prevalence estimates by examining physicians’ awareness of DM, self-rated understanding of the law, attitudes toward malpractice litigation, and their associations with defensive practices. In this sample, defensive behaviors were common despite limited formal awareness of DM and notable gaps in legal literacy. Overall attitudes toward malpractice litigation were not independently associated with DM scores, whereas perceiving litigation as a personal attack was independently associated with higher defensive engagement. Taken together, these findings suggest that threat-specific perceptions may be more closely associated with defensive practices than broader attitudes toward litigation. Figure 1 summarizes the main adjusted associations together with the broader contextual considerations discussed below. These interpretations should be viewed in the context of a convenience sample weighted toward younger, resident, and hospital-based physicians, and may not fully reflect the experience of more senior, private-sector, or non-hospital physicians.

The high prevalence of self-reported DM observed in this study is broadly consistent with reports from both Middle Eastern and non-Middle Eastern settings [7, 26, 33–36]. However, the Jordanian pattern appears somewhat distinct. Self-reported defensive behavior was widespread despite only low-to-moderate overall intensity and limited formal medicolegal awareness. This differs from much of the North American literature, where DM is often framed in relation to malpractice premiums, claim frequency, and specialty-specific litigation exposure [10, 11]. In this sample, legal uncertainty, patient expectations, uneven legal literacy, and limited institutional support appeared to be at least as relevant as formal litigation risk. Because the sample was weighted toward younger, hospital-based physicians who may be more exposed to high-volume, high-pressure settings, the relative importance of these contextual factors may differ in the broader Jordanian physician workforce. The predominance of assurance behaviors, particularly additional diagnostic testing, is consistent with prior literature suggesting that such practices may be easier to justify clinically while contributing to overuse and downstream costs [6, 17, 37–39]. Some reported assurance behaviors may also reflect cautious but clinically appropriate practice under uncertainty rather than objectively unnecessary care. Importantly, the DM measure reflects physicians’ perceptions of precautionary or defensive care rather than objectively verified overuse. Reported variation in DM may therefore reflect differences in clinical judgment, risk tolerance, and awareness, in addition to actual differences in practice.

A non-linear pattern was observed between specialty-risk grouping and DM intensity, although this finding should be interpreted cautiously. Differences were observed in bivariate analysis, but only one adjusted post-hoc comparison remained significant, and specialty-risk category was not independently associated with DM in the multivariable model. This pattern should be considered exploratory rather than evidence of a definitive low-risk paradox. Specialty-based malpractice risk alone may not capture the practice conditions most closely associated with reassurance-oriented behavior in Jordan.

Physicians in low-risk settings may still work under conditions associated with diagnostic uncertainty, high patient volume, and limited access to specialist support [40–44]. Patient-related pressure may be part of this broader context. In this study, many physicians reported pressure to provide care that they perceived as potentially unnecessary. Prior research also links patient expectations and time constraints to reassurance-oriented practice, particularly in busy frontline settings [17, 45, 46]. In Jordan, these pressures may be especially relevant in public-sector and primary-care settings where continuity of care and formal risk-management support may be limited [21, 47]. These observations are best viewed as contextual interpretations rather than direct findings of the adjusted models.

One of the most important findings is the apparent legal literacy gap. Several years after the enactment of the 2018 Medical Liability Law, most physicians reported poor self-rated understanding of the law, and fewer than one-third had heard the formal term “defensive medicine”. Because this measure was self-reported, it reflects perceived rather than objective legal knowledge. Even so, the findings suggest a gap between legal reform and physicians’ day-to-day understanding of its implications, a concern also reported in comparable settings [20, 48]. The practical implications operate at two complementary levels. First, educational interventions could include applied medicolegal teaching in undergraduate curricula, residency orientation, and accredited continuing medical education, supported by case-based guidance on the practical application of the 2018 Medical Liability Law. Second, system-level responses could include clearer institutional guidance, accessible legal consultation, and stronger legal and risk-management support within healthcare organizations. Professional bodies such as the Jordan Medical Council and Jordan Medical Association could help support dissemination through structured training and guidance materials. These suggestions should be viewed as context-specific priorities for further evaluation rather than as established solutions.

Given the overrepresentation of early-career physicians in this sample, these concerns may be particularly relevant to residency training programs. Structured medicolegal orientation during residency, together with accessible supervisory and institutional support mechanisms, may help reduce uncertainty around malpractice and limit defensively motivated practice.

The attitudinal findings highlight an important psychological dimension. Physicians appeared to hold generally balanced views of malpractice litigation, recognizing both its emotional burden and its potential role in accountability, consistent with prior work [23]. Because items 1–3 were reverse-coded, higher attitude scores reflect less negative and more accepting views of malpractice litigation. However, overall attitude scores were not independently associated with DM, whereas perceiving litigation as a personal attack was associated with higher defensive engagement. Overall, threat-specific appraisals may be more closely associated with defensive practices than broader evaluative views, although temporal direction cannot be determined from this cross-sectional design. Greater familiarity with DM was also associated with higher attitude scores, although the direction of this association cannot be determined. In addition, higher DM scores were independently associated with lower attitude scores. Together, these results suggest that the relationship between defensive practice and litigation attitudes is not uniform and may depend on the specific attitudinal dimension considered.

Younger physicians and those with personal experience of malpractice litigation had higher DM scores. These associations may reflect differences in perceived professional vulnerability, although this was not directly measured. Prior studies suggest that early-career physicians may experience greater uncertainty and may feel more vulnerable to complaints or litigation [26, 33]. The association with litigation experience is also consistent with literature describing psychological distress after medicolegal events [49–51], which may in turn be associated with more precautionary clinical behavior. However, because the sample overrepresents younger and early-career physicians, these associations may partly reflect sample composition rather than broader population-level patterns. The adjusted models explained only a modest proportion of the variance. These variables should therefore be interpreted as partial correlates rather than strong explanatory determinants of DM or malpractice-related attitudes. Younger physicians also showed higher attitude scores in bivariate analysis, but this did not persist after adjustment and should therefore be interpreted cautiously. Similarly, the association with female sex did not persist after adjustment.

Perceived financial and institutional vulnerability may represent another contextually relevant consideration in this setting. More than half of the participants lacked liability insurance, and only a minority felt adequately protected. This is consistent with international evidence suggesting that litigation exposure and inadequate professional liability protection may be associated with precautionary practice, potentially through financial anxiety, professional isolation, and uncertainty about available support [52–54]. However, because insurance adequacy was not independently associated with DM after adjustment, these findings are best interpreted as descriptive indicators of perceived vulnerability rather than as independent explanatory factors. Because private-sector and more senior physicians, who may have different insurance arrangements, were underrepresented in this sample, the actual prevalence and implications of insurance gaps in the broader Jordanian physician workforce may differ from what is observed here. In Jordan, these observations point to the potential value of clearer standards for liability coverage and improved institutional support. For residents and early-career physicians, institution-based coverage models and post-incident support mechanisms may be particularly relevant.

This study has important strengths. It is among the first to assess DM in Jordan following the 2018 Medical Liability Law while examining defensive practices alongside attitudes, litigation experience, insurance adequacy, and perceived legal understanding. The use of structured instruments and multivariable analyses provides a broader view of defensive behaviors within this sample. At the same time, the modest explanatory power of the adjusted models suggests that additional individual, organizational, and cultural influences are also likely to be associated with both DM and malpractice-related attitudes in this setting.

Several limitations should be considered when interpreting these findings. First, the use of convenience sampling, predominantly through online recruitment and supplemented by paper-based questionnaires in selected hospitals, introduces potential selection, coverage, and volunteer bias. Because the survey link was openly shared, a conventional response rate could not be calculated. The sample was weighted toward younger, resident, and hospital-based physicians. Although this partly reflects the relatively young physician workforce in Jordan, publicly available data from the Jordan Medical Association indicate that the largest physician age group nationally is 30–39 years, suggesting that physicians younger than 30 years remain overrepresented in the present sample relative to the national workforce. The observed prevalence and intensity of self-reported defensive practices may therefore have been somewhat overestimated. Because younger age was independently associated with higher DM scores in the adjusted analyses, the overrepresentation of younger physicians may have contributed directly to this potential overestimation. In addition, physicians with stronger opinions about medicolegal issues or greater personal exposure to malpractice-related concerns may have been more likely to participate, potentially inflating both DM reporting and the apparent medicolegal literacy gap. Differences in recruitment mode may also have introduced response bias, as physicians who completed the online questionnaire may have differed systematically from those who completed the paper version in age, experience, and practice setting. Accordingly, the findings should not be generalized uncritically to the broader Jordanian physician workforce, particularly older, private-sector, rural, and non-hospital physicians, who were underrepresented.

Second, DM was assessed using self-reported measures based on physicians’ perceptions of “precautionary or defensive care”, without objective validation through chart review, utilization data, or claims data. The findings should therefore be interpreted as reflecting perceived rather than objectively verified defensive practices. This introduces potential recall bias, subjective interpretation, and social desirability bias. It may also have resulted in either over-reporting or under-reporting, depending on individual clinical judgment and risk tolerance. Similarly, self-rated understanding of the 2018 Medical Liability Law reflects perceived rather than objectively measured legal knowledge.

Third, although the questionnaire underwent forward-backward translation and pilot testing with minor contextual adjustments, it was not formally validated as a Jordan-specific psychometric instrument. It should therefore be interpreted as a context-adapted tool rather than a fully validated local measure. This limitation is particularly relevant to the interpretation of the attitude constructs, as medicolegal perception items may not transfer fully across linguistic and cultural settings without formal local validation. Accordingly, findings related to attitude constructs should be interpreted with appropriate caution. Future studies should formally validate the Arabic-translated instrument in the Jordanian setting, including assessment of construct validity, dimensionality, and test-retest reliability.

Fourth, the cross-sectional design precludes causal inference. Observed associations between demographics, experiential, and attitudinal factors and DM should therefore not be interpreted as directional relationships. Residual confounding also cannot be excluded.

Fifth, the specialty-risk classification was a pragmatic, a priori categorization adapted from prior literature rather than a formally validated Jordan-specific risk model; findings related to specialty differences should therefore be considered exploratory and hypothesis-generating.

Finally, the multivariable models explained only a modest proportion of the variance. The exploratory stepwise analyses should therefore be interpreted as supplementary and hypothesis-generating rather than definitive. The relatively modest R² values indicate that the identified variables account for only part of the observed variation in DM and attitudes and that additional unmeasured influences are likely to be important. Future research should include larger, more representative samples, incorporate objective utilization or claims-based measures where feasible, and consider longitudinal, qualitative, or mixed-methods designs to better understand underlying mechanisms and inform context-specific interventions.

## Conclusions

DM was common in this sample of physicians in Jordan and was characterized by low-to-moderate intensity and a predominance of assurance-type behaviors. In this sample, defensive practices appeared to be more closely associated with perceived professional vulnerability, limited perceived medicolegal understanding, contextual practice pressures, and threat-related interpretations of litigation than with specialty-specific litigation risk alone. These findings support context-specific responses, including strengthening practical medicolegal education, improving access to professional liability coverage and institutional legal support, and addressing the psychological burden associated with complaints and litigation. The modest explanatory power of the adjusted models further suggests that additional individual, organizational, and cultural determinants of defensive practices likely remain unmeasured. Because the study was based on a convenience sample that overrepresented younger, resident, and hospital-based physicians, the findings should be interpreted as exploratory and should not be generalized uncritically to the wider Jordanian physician workforce. Larger and more representative studies are needed to confirm these associations and inform policy and practice in Jordan.

## Supplementary Information

Below is the link to the electronic supplementary material.

Supplementary Material 1

Supplementary Material 2

## Data Availability

All relevant data are within the manuscript and its Supporting Information files. Additional de-identified data underlying the findings of this study are available from the corresponding author upon reasonable request, in accordance with institutional data protection policies.

## Abbreviations

DM: Defensive Medicine
SD: Standard Deviation
IQR: Interquartile Range
SE: Standard Error
CI: Confidence Interval
