# Supplementary material for: Defensive medicine practices and attitudes toward malpractice among physicians in Jordan: a cross-sectional study
Source: BMC Health Serv Res. 2026 Jun 30;26:1288. doi: 10.1186/s12913-026-15053-5 (PMC13591804; doi:10.1186/s12913-026-15053-5)
Supplement: Supplementary file 1 [file 12913_2026_15053_MOESM1_ESM.pdf]

# The Prevalence of Defensive Medicine and Its Triggering factors Among Physicians In Jordan: A Cross-Sectional Study

**This study has obtained the ethical approval from IRB committee at Jordan University of Science and Technology , and it's conducted under the supervision of Dr. Heba Ghozlan.**

## Section 1 : Sociodemographic characteristics

- **Gender :(Male – Female)**
- **Age :________**
- **Current medical position/level:_________**
- **Sector of service (Type of hospital):______**
- **Years of experience (total length of time in the profession since graduation from medical school):_________**
- **Medical specialty:________**

**Total working time practicing medicine (hours per week)**

○ < 72 hours / week

○ 72 - 130 hours / week

○ > 130 hours / week

**Area of service** *

○ North of Jordan (Jerash, Irbid, Mafraq, Ajloun)

○ Central Jordan (Amman, Zarqa, Balqa, and Madaba)

○ South of Jordan (Karak, Tafila, Ma'an, Aqaba)

**If you are a resident : Are you on paid or unpaid program?**

○ Paid

○ Unpaid

○ I am not a resident

## Section 2 : Measuring the practice of Defensive Medicine

- This section contains 10 items which aim to understand the prevalence of defensive behaviors among Jordanian physicians.
- Questions in this section can be answered by either (Never , Rarely , Sometimes , Usually , Always).

| | Items | Never | Rarely | Sometimes | Usually | Always |
|---|---|---|---|---|---|---|
| 1 | Asking for more screenings than necessary | | | | | |
| 2 | Asking for more diagnostic tests ( Lab, Imaging, etc.) than necessary | | | | | |
| 3 | Asking for unnecessary consultations | | | | | |
| 4 | Hospitalizing the patient without necessary | | | | | |
| 5 | Performing invasive operation (like biopsy) unnecessary | | | | | |
| 6 | Prescribing unnecessary medication to the patient | | | | | |
| 7 | Taking initiative less frequently | | | | | |
| 8 | Referring the patient to other department / center / hospital | | | | | |
| 9 | Avoiding treating risky patients | | | | | |
| 10 | Avoiding new treatment methods | | | | | |

## Section 3 : Awareness regarding Defensive Medicine and malpractice litigation

Have you heard about the concept of Defensive Medicine before?

- ○ Yes
- ○ No

**Do you think that malpractice litigation cases have increased in the recent years?**

- Yes
- No

**Do you completely understand the Jordanian medical liability law established in 2018?**

- Yes
- No

## Section 4 : Triggering factors to practice Defensive Medicine

**Have you ever experienced lawsuits because of malpractice?**

- Yes
- No

**Has any of your colleagues been exposed to lawsuits because of malpractice?**

- Yes
- No
- I don't know

**In your opinion, is your liability insurance adequate?**

- Yes
- No
- I don’t have liability insurance

## Section 5 : Attitude toward malpractice litigations

- This section aims to assess the attitude of Jordanian physicians toward malpractice litigations.
- Please answer the following questions according to your feeling of the proposed situation about the potential consequences of malpractice litigations.

→ In your opinion , The potential consequences of malpractice litigations are :

| | items | Strongly disagree | Disagree | Neutral | Agree | Strongly agree |
|---|---|---|---|---|---|---|
| **1** | Are harmful for the reputation and self-confidence of a physician | | | | | |
| **2** | Make me stressed and Evoke in me a feeling of guilt | | | | | |
| **3** | I regard as a personal attack | | | | | |
| **4** | I regard as a form of justice | | | | | |
| **5** | Are a way to improve the quality of care | | | | | |

## Section 6 : Patient's perceived pressure demanding for care

**when patients request further examinations or treatments , I feel pressured to ————— even if it is clinically not strictly necessary (choose all that apply) :**

- ☐ Do further examination and treatment
- ☐ Prescribe medications
- ☐ Refer the patient to other center
- ☐ I don't feel pressured to do anything

---

Thank You So Much
