# Supplementary material for: Defensive medicine practices and attitudes toward malpractice among physicians in Jordan: a cross-sectional study
Source: BMC Health Serv Res. 2026 Jun 30;26:1288. doi: 10.1186/s12913-026-15053-5 (PMC13591804; doi:10.1186/s12913-026-15053-5)
Supplement: Supplementary file 2 [file 12913_2026_15053_MOESM2_ESM.docx]

**Supplementary Material**

**Supplementary Table S1.** Distribution of Physician Specialties (n = 211)

| Specialty | n | % |
| --- | --- | --- |
| General Practitioner (GP) | 35 | 16.6 |
| General surgery | 35 | 16.6 |
| General Internal medicine | 18 | 8.5 |
| Anesthesia | 17 | 8.1 |
| Pediatrics | 13 | 6.2 |
| Dermatology | 12 | 5.7 |
| Family medicine and preventive medicine | 10 | 4.7 |
| Orthopedics and Traumatology | 9 | 4.3 |
| ENT | 8 | 3.8 |
| Neurosurgery | 8 | 3.8 |
| Urology | 8 | 3.8 |
| Radiology and nuclear medicine | 7 | 3.3 |
| Obstetrics and gynecology | 6 | 2.8 |
| Neuromedicine | 5 | 2.4 |
| Ophthalmology | 5 | 2.4 |
| Internship doctor | 4 | 1.9 |
| Psychiatry | 4 | 1.9 |
| Cardiology | 2 | 1.0 |
| Clinical pathology | 1 | 0.5 |
| Endocrinology | 1 | 0.5 |
| Forensic Medicine | 1 | 0.5 |
| Nephrology | 1 | 0.5 |
| Oncology | 1 | 0.5 |
| Total | **211** | **100.0** |

Percentages may not total exactly 100 due to rounding.

**Supplementary Table S2.** Respondents by Specialty Risk Category

| Risk Category | Specialties |
| --- | --- |
| ****High-Risk**** | General Surgery, Orthopedics and Traumatology, Neurosurgery, Obstetrics and Gynecology, Anesthesia |
| ****Moderate-Risk**** | Pediatrics, General Internal Medicine, Dermatology, ENT, Urology, Radiology and Nuclear Medicine, Neuromedicine, Cardiology, Ophthalmology |
| ****Low-Risk**** | General Practitioner (GP), Family Medicine and Preventive Medicine, Psychiatry, Internship Doctor, Clinical Pathology, Endocrinology, Forensic Medicine, Nephrology, Oncology |

Specialties were categorized into high-, moderate-, and low-risk groups using a pragmatic, a priori classification informed by international malpractice litigation patterns (Jena, Seabury et al. 2011), and adapted to the Jordanian medico-legal context. Pediatrics and dermatology were classified as moderate-risk in this study as part of this contextual adaptation.

**Supplementary Table S3.** Defensive Medicine Scores by Physician Characteristics

| Factor Group | Specific Factor | Level | *n* | Median (IQR) |
| --- | --- | --- | --- | --- |
| Demographics | Gender | Male | 118 | 20.5 (17-24) |
|  |  | Female | 93 | 21 (17-24) |
|  | Age | Continuous | 211 | - |
| Professional | Specialty Risk | Low | 58 | 22 (18-25) |
|  |  | Moderate | 78 | 19 (15-23) |
|  |  | High | 75 | 21 (18-24) |
|  | Professional Experience | Continuous | 211 | - |
|  | Sector | University Hospitals | 94 | 21 (17-24) |
|  |  | Royal Medical Services | 50 | 20 (17-23) |
|  |  | Ministry of Health | 41 | 20 (18-24) |
|  |  | Private Sector | 26 | 21.5 (20-25) |
|  | Geographic Region | North | 145 | 21 (17-24) |
|  |  | Central | 60 | 21 (17-24) |
|  |  | South | 6 | 21.5 (16-24) |
| Awareness | Heard of DM | No | 146 | 21 (17-24) |
|  |  | Yes | 65 | 20 (17-24) |
|  | Perceived Increase in Malpractice Litigation | No | 27 | 22 (16-27) |
|  |  | Yes | 184 | 20 (17-24) |
|  | **Self-Rated Understanding of the 2018 Medical Liability Law** | Continuous | 211 | - |
| Triggering Factors | Personal Lawsuits | No | 195 | 20 (17-24) |
|  |  | Yes | 16 | 23.5 (21-31.5) |
|  | Colleagues' Lawsuits | No | 129 | 20 (17-24) |
|  |  | Yes | 82 | 21 (17-24) |
|  | Insurance Adequacy | Absent | 108 | 21 (18-24 |
|  |  | Inadequate | 60 | 20.5 (16.5-26) |
|  |  | Adequate | 43 | 20.0 (16-22) |

Continuous variables were analyzed using Spearman's rank correlation coefficient; median values are not applicable for these rows.

**Supplementary Table S4.** Post-hoc Pairwise Comparisons of Defensive Medicine Scores by Specialty Risk Group Using Dwass, Steel, Critchlow-Fligner Method

| Comparison | DSCF Value | p-value |
| --- | --- | --- |
| Low-risk vs Moderate-risk | 3.3736 | 0.0449 |
| Moderate-risk vs High-risk | 2.8846 | 0.1028 |
| Low-risk vs High-risk | 0.8717 | 0.8112 |

Dwass, Steel, Critchlow-Fligner (DSCF) Method was performed for pairwise comparisons following a significant overall difference across specialty risk groups.

**Supplementary Table S5.** Attitude Scores by Physician Characteristics

| Factor Group | Specific Factor | Level | *n* | Median (IQR) |
| --- | --- | --- | --- | --- |
| Demographics | Gender | Male | 118 | 15 (14-16) |
|  |  | Female | 93 | 16 (14-17) |
|  | Age | Continuous | 211 | - |
| Professional | Specialty Risk | Low | 58 | 16 (14-17) |
|  |  | Moderate | 78 | 15 (14-16) |
|  |  | High | 75 | 15 (14-17) |
|  | Professional Experience | Continuous | 211 | - |
|  | Sector | University Hospitals | 94 | 16 (15-17) |
|  |  | Royal Medical Services | 50 | 15 (14-16) |
|  |  | Ministry of Health | 41 | 15 (14-16) |
|  |  | Private | 26 | 15 (14-16) |
|  | Geographic Region | North | 145 | 15 (14-17) |
|  |  | Central | 60 | 15(14-16) |
|  |  | South | 6 | 14 (13-16) |
| Awareness | Heard of DM | No | 146 | 15 (14-16) |
|  |  | Yes | 65 | 16 (15-17) |
|  | Perceived Increase in Malpractice Litigation | No | 27 | 15 (14-17) |
|  |  | Yes | 184 | 15 (14-16) |
|  | **Self-Rated Understanding of the 2018 Medical Liability Law** | Continuous | 211 | - |
| Triggering Factors | Personal Lawsuits | No | 195 | 15 (14-16) |
|  |  | Yes | 16 | 15 (14-16) |
|  | Colleagues' Lawsuits | No | 129 | 15 (14-16) |
|  |  | Yes | 82 | 16 (14-17) |
|  | Insurance Adequacy | Absent | 108 | 15 (14-16) |
|  |  | Inadequate | 60 | 15 (14-16) |
|  |  | Adequate | 43 | 16 (15-17) |

Continuous variables were analyzed using Spearman's rank correlation coefficient; median values are not applicable for these rows.

**Table S6. Effect Sizes for Bivariate Comparisons (N = 211)**

| Factor Group | Specific Factor | Effect Size (DM) | Magnitude (DM) | Effect Size (Attitude) | Magnitude (Attitude) |
| --- | --- | --- | --- | --- | --- |
| Demographics | Gender | *d* = 0.005 | negligible | *d* = 0.166 | small |
|  | Age | *ρ* = –0.230 | small | *ρ* = –0.150 | small |
| Professional | Specialty risk | *η*² = 0.033 | small | *η*² = 0.006 | negligible |
|  | Professional experience | *ρ* = –0.113 | negligible | *ρ* = –0.131 | negligible |
|  | Sector | *η*² = 0.016 | negligible | *η*² = 0.024 | negligible |
|  | Geographic region | *η*² = 0.001 | negligible | *η*² = 0.027 | negligible |
| Awareness | Heard of defensive medicine | *d* = 0.040 | negligible | *d* = 0.143 | small |
|  | Perceived increase in malpractice litigation | *d* = 0.046 | negligible | *d* = 0.039 | negligible |
|  | Self‑rated understanding of the 2018 Medical Liability Law | *ρ* = –0.028 | negligible | *ρ* = –0.070 | negligible |
| Triggering factors | Personal lawsuit experience | *d* = 0.181 | small | *d* = 0.087 | negligible |
|  | Colleague’s lawsuit experience | *d* = 0.011 | negligible | *d* = 0.059 | negligible |
|  | Insurance adequacy | *η*² = 0.012 | negligible | *η*² = 0.019 | negligible |

Effect sizes: for Mann‑Whitney *U*, Cohen’s d = z/√N; for Kruskal‑Wallis, η² = *H*/(N‑1); for Spearman, *ρ*. Thresholds: *d* or *ρ*: 0.2 (small), 0.5 (medium), 0.8 (large); *η*²: 0.01 (small), 0.06 (medium), 0.14 (large). Non‑significant associations (*p* ≥ 0.05 in Table 7) all had negligible effect sizes (*d* < 0.2, η² < 0.01, |*ρ*| < 0.1). N = 211 for all comparisons.

**Supplementary Table S7. Multivariable Linear Regression Analysis of Factors Associated with Defensive Medicine Score (N = 211)**

| Variable | *B* | SE | 95% CI | p-value |
| --- | --- | --- | --- | --- |
| Intercept | 30.47 | 2.15 | 26.23, 34.70 | <0.001* |
| Age | -0.19 | 0.06 | -0.30, -0.08 | <0.001* |
| Personal litigation experience | 4.91 | 1.31 | 2.34, 7.49 | <0.001* |
| Perception of litigation as personal attack | -1.26 | 0.35 | -1.96, -0.56 | <0.001* |

Stepwise multiple linear regression model for defensive medicine score (final model with 3 predictors). Model summary: F(3, 207) = 13.45, p < 0.001, R² = 0.163, adjusted R² = 0.151, Root MSE = 4.98. Variables were selected using stepwise selection (entry/stay criterion p < 0.15). Coefficients are unstandardized (B). Statistically significant coefficients (p < 0.05) are indicated with *.

**Supplementary Table S8. Multivariable Linear Regression Analysis of Factors Associated with Attitude Toward Malpractice Litigation (N = 211)**

| Variable | *B* | SE | 95% CI | p-value |
| --- | --- | --- | --- | --- |
| Intercept | 18.79 | 1.11 | 16.60, 20.98 | <0.001 |
| Sex | 0.45 | 0.31 | -0.16, 1.06 | 0.149 |
| Age | -0.08 | 0.03 | -0.13, -0.03 | **0.001*** |
| Familiarity with defensive medicine | 0.79 | 0.33 | 0.13, 1.44 | **0.019*** |
| Personal litigation experience | -0.88 | 0.59 | -2.05, 0.29 | 0.141 |
| DM score | -0.06 | 0.03 | -0.12, -0.01 | **0.030*** |

Stepwise regression model for attitude score (final model with 5 predictors). Model summary: F(5, 205) = 5.04, p = 0.0002, R² = 0.109, adjusted R² = 0.088, Root MSE = 2.17. Variables were selected using stepwise selection from the full multivariable model (entry/stay p < 0.15). Coefficients are unstandardized (B). Statistically significant coefficients (*p* < 0.05) are indicated with *.

**References**

Jena, A. B., S. Seabury, D. Lakdawalla and A. Chandra (2011). "Malpractice risk according to physician specialty." N Engl J Med **365**(7): 629-636.
